# Synthesis and *In Vitro* Antimicrobial and Anthelminthic Evaluation of Naphtholic and Phenolic *Azo* Dyes

**DOI:** 10.1155/2020/4850492

**Published:** 2020-06-01

**Authors:** Joseph Kwasi Adu, Cedric Dzidzor Kodjo Amengor, Nurudeen Mohammed Ibrahim, Cynthia Amaning-Danquah, Charles Owusu Ansah, Dorcas Dzifa Gbadago, Joseph Sarpong-Agyapong

**Affiliations:** ^1^Department of Pharmaceutical Chemistry, Faculty of Pharmacy and Pharmaceutical Sciences, College of Health Sciences, Kwame Nkrumah University of Science and Technology (KNUST), Kumasi, Ghana; ^2^Department of Pharmaceutical Chemistry, School of Pharmacy, University of Health and Allied Sciences, Ho, Ghana; ^3^Department of Pharmacology, Faculty of Pharmacy and Pharmaceutical Sciences, College of Health Sciences, Kwame Nkrumah University of Science and Technology (KNUST), Kumasi, Ghana

## Abstract

The antimicrobial activity of 2-naphtholic and phenolic *azo* compounds was determined against seven microbial species, *Staphylococcus aureus* (ATCC 25923), *Streptococcus pyrogenes* (clinical), and *Enterococcus faecalis* (ATCC 29212), *Salmonella typhi* (clinical), *Pseudomonas aeruginosa* (ATCC 27853), *Escherichia coli* (ATCC 251922), and *Candida albicans* (ATCC 10231), using the high-throughput spot culture growth inhibition assay (HT-SPOTi). The minimum inhibitory concentrations (MIC) were determined for the active *azo* dyes. All the *azo* compounds (A1–B4) were screened for anthelmintic activity against adult Ghanaian earthworms, *Hyperiodrilus* spp. As part of the systematic investigation for biological activity, all the *azo* compounds exhibited good antimicrobial activity against the seven human pathogenic microorganisms. All the compounds exhibited anthelminthic activity against adult Ghanaian earthworms, *Hyperiodrilus* spp.

## 1. Introduction

Over the past decades, the emergence of resistant pathogens in humans has been on the ascendency and has become a global health concern in chemotherapy of infectious diseases [1]. This has therefore stimulated efforts in the field of antimicrobial chemotherapy search for novel drugs to curtail this menace [2]. Bacterial resistance is an inevitable consequence of evolution, and without continued replacement of current antibacterial agents, humanity runs the risk of returning to a preantibiotic era [3]. A section of neglected tropical diseases are associated with helminths, and they are amongst the most common ones affecting the world and posing major public health threat, thus contributing to high morbidity and mortality [3, 4]. The existence of few antimicrobials and anthelminthics coupled with current chemotherapy losing their efficacy calls for continuous research for new but more effective compounds in the drug discovery industry [5, 6].

**Figure 1:**
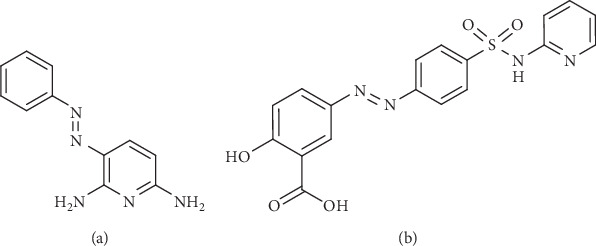
Chemical structures of current azo-based drugs: (a) phenazopyridine and (b) sulfasalazine.

With regards to this assertion, *azo* compounds have been found to be useful, extending the scope of drug design and allowing different mechanisms of action [7, 8]. The increasing interest in the development of *azo* compounds is due to their functions and versatile biological activities including antimicrobial, anti-inflammatory, anthelminthic, antiviral, and anticancer effects [9, 10]. Typical examples of *azo* dye compounds which are drugs include phenazopyridine, which is a urinary tract analgesic, and sulfasalazine for inflammatory bowel disease (Figure 1).

**Scheme 1:**
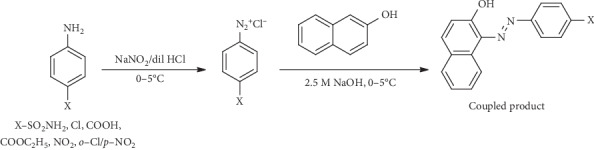
General reaction for the synthesis of the naphtholic *azo* compounds [12].

Although they are synthetically feasible by simple standard diazotisation and coupling reactions, there is still more to be carried out particularly with regards to diversification of functional groups [11]. Following on this trajectory, we present herein our findings on the synthesis and *in vitro* antimicrobial and anthelminthic evaluation of naphtholic and phenolic *azo* dyes derived from the coupling of various primary aromatic amines with 2-naphthol and phenol.  Scheme 1 represents a general synthetic pathway for the *azo* dyes with their proposed names. The eight *azo* compounds are [A1] (*E*)-4-((2-hydroxynaphthalen-1-yl) diazenyl) benzoic acid, [A2] (*E*)-1-((4-chlorophenyl) diazenyl) naphthalen-2-ol, [A3] (*E*)-ethyl 4-((2-hydroxynaphthalen-1-yl) diazenyl) benzoate, [A4] (*E*)-4-((2-hydroxynaphthalen-1-yl) diazenyl) benzene sulphonamide, [B1] (*E*)-4-((4-hydroxyphenyl) diazenyl) benzoic acid, [B2] (*E*)-ethyl 4-((4-hydroxyphenyl) diazenyl) benzoate, [B3] (*E*)-4-((2-chloro-4-nitrophenyl) diazenyl) phenol, and [B4] (*E*)-4-(4-nitrophenyl) diazenyl) phenol. These compounds are coded based on their reacting components and are shown in Table 1. The synthesised *azo* compounds were then investigated for their antimicrobial and anthelminthic activities.

## 2. Materials and Methods

### 2.1. Chemistry and Instrumentation

All reagents and solvents used in this present study were obtained from BDH chemicals as analytical or technical grade. Reaction progress was monitored using thin layer chromatography, which was performed by employing precoated silica gel plate (Merck F_254_) and visualised with UV light (254 nm and 357 nm) or iodine vapour where necessary. Melting point data of the synthesised compounds were obtained by using one end or open capillary tubes on a Gallenkamp melting point apparatus (England) and are uncorrected. Compounds were purified by recrystallisation from suitable solvents appended to synthetic data in Table 1. Infrared (IR) spectra were recorded using FTIR PerkinElmer in the range 400–4000 cm^−1^. Ultraviolet-visible (UV-vis) spectra were measured on a PerkinElmer spectrophotometer at 200–800 nm in methanol.

### 2.2. Synthesis of the *Azo* Compounds

**Scheme 2:**
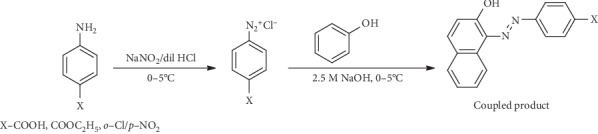
General reaction for the synthesis of the phenolic *azo* compounds [12].

The synthesis of naphtholic and phenolic *azo* compounds is presented in Schemes 1 and 2.

Into a round-bottom flask (50 mL) equipped with a magnetic stirring bar, a mixture of primary aromatic amine (10 mmol) and concentrated HCl (36% w/v) was stirred until a clear solution was obtained. This solution was cooled to 0°C–5°C, and a solution of sodium nitrite in water (10 ml) was then added dropwise maintaining the temperature below 5°C. The diazonium salt solution was covered and kept in the ice bath. Starch iodide paper was used to test for the formation of diazonium salt-spontaneous formation of blue-black coloration. The diazonium salt was used immediately for the coupling reaction.

#### 2.2.1. Step II: Coupling Procedure

Into a round-bottom flask (50 mL), the corresponding 2-naphthol or phenol (10 mmol) was dissolved in NaOH (10% w/v, 50 ml) and cooled to 0°C–5°C in an ice bath. This solution was then gradually added to the cooled diazonium salt solution, and the resulting mixture was stirred at 0°C–5°C for 1 h. The resulting crude precipitate was filtered, washed several times with cold water, and recrystallized from appropriate solvent to yield the final *azo* compound [12] (Table 1).

### 2.3. Antimicrobial Activity

#### 2.3.1. Culture Media and Reference Antibiotics

Nutrient agar and broth as well as Saboraud dextrose agar and broth were purchased from Oxoid Limited, Basingstoke, United Kingdom. Ciprofloxacin, cefuroxime, amoxycillin, and clotrimazole were obtained from Sigma Aldrich™ (Michigan, USA).

### 2.4. Source of Test Organisms

Gram-positive bacteria, Gram-negative bacteria, and fungi were used, including five type strains of bacteria and five clinical strains. Gram-negative bacteria included *Escherichia coli* (ATCC 25922), *Pseudomonas aeruginosa* (ATCC 27853), and *Salmonella typhi*. Gram-positive bacteria included *Staphylococcus aureus* (ATCC 25923) and *Streptococcus pneumoniae* (ATCC 49691). Clinical fungal strain included *Candida albicans* (ATCC 10231). The test organisms were all obtained from the Department of Biological Sciences, Kwame Nkrumah University of Science and Technology (KNUST), Kumasi, Ghana. They were maintained on nutrient agar slants containing 30% glycerol and stored at −4°C in a frost-free freezer in the Microbiology laboratory section at the Department of Pharmaceutics, Kwame Nkrumah University of Science and Technology (KNUST), Kumasi, Ghana.

#### 2.4.1. Step I: Preparation of the Standard 96-Well Plates

An amount of agar medium was melted in a steam bath or in a microwave oven. It was placed in a water bath and kept at a temperature of 55°C–60°C to prevent it from solidifying. A stock concentration of 50 mg/ml was prepared initially using methanol in a polymerase chain reaction (PCR) half-skirted 96-well plate to give a wide concentration range. Eleven different concentrations of *azo* compounds and reference drugs (ciprofloxacin, cefuroxime, amoxicillin, and fluconazole) were prepared using serial dilution. Each compound (2 *μ*L) and reference drug dilution was pipetted and transferred into their corresponding wells in a standard 96-well plate, and the melted agar (200 *μ*L) was dispensed. The well-plates were then shaken for 10 s to mix thoroughly. After the agar medium was dispensed, the plates were left in a safety cabinet undisturbed to solidify [13].

#### 2.4.2. Step II: Spotting and Incubation of Prepared Well Plates

Bacterial suspension (2 *μ*L; 1 × 10^6^ cfu/ml) was pipetted with a micropipette on to each well of the 96-well plate (each microorganism per plate) and allowed it to be absorbed into the agar for 5 min. The plates were sealed with parafilm and wrapped with aluminium foil. The plates were incubated inverted at 37°C. The growth was observed after 24 hrs. The well containing the lowest concentration of a compound for which no growth is observed and recorded within the incubation period is determined as the MIC (minimum inhibitory concentration) of that compound against the organisms [13].

#### 2.4.3. Step III: Minimum Inhibitory Concentration (MIC) Determination

Different concentrations of the *azo* compounds were tested against a panel of Gram-positive bacteria (*Staphylococcus aureus*, *Enterococcus faecalis*, and *Streptococcus pyogenes*), Gram-negative bacteria (*Salmonella typhi*, *Escherichia coli*, and *Pseudomonas aeruginosa*), and a fungus (*Candida albicans*) to determine their minimum inhibition concentrations (MICs) using the high-throughput spot-culture growth inhibition assay (HT-SPOTi).

In the HT-SPOTi assay, molten agar maintained at 55°C–60°C was dispensed into 96-well plates to which 2 *μ*L of serially diluted *azo* compounds have been added starting from a stock of 50 mg/mL. Bacterial suspension (2 *μ*L; 1 × 10^6^ cfu/ml) was added to each plate sealed and incubated for 18–24 h. The lowest concentration at which bacterial growth was completely inhibited by the compound was observed visually, and the MIC was recorded. The reference drugs used were ciprofloxacin, cefuroxime, amoxicillin, and fluconazole [13].

### 2.5. Anthelminthic Activity

#### 2.5.1. Reagents and Reference Antibiotics

The reagents used included methanol (BDH Chemicals), dimethyl sulphoxide (DMSO) (BDH Chemicals) (ACS grade ≥ 99%), and piperazine citrate (Sigma Aldrich).

The procedure for screening for the anthelminthic activity was with reference to Raghavendra and Kumar [14]. The anthelminthic activity was carried out on adult Ghanaian worms, *Hyperiodrilus* spp. The worms were collected from a local moist habitat and prior assay. They were washed with water to remove dirt, soil, and faecal matter from the skin of the worm. They were divided into groups in three Petri dishes with each containing three adult worms. Dimethyl sulphoxide (DMSO, 0.5% v/v) was used as the control, while piperazine citrate was used as a reference standard for this assay. Concentrations of 0.625, 1.25, 2.5, and 5 mg/ml were prepared for each of the *azo* compounds and the standard drug solutions. Three adult worms (triplicate determination) were then introduced into the Petri dishes containing solutions of the *azo* compounds and the standard drug. The worms in the Petri dishes were observed, and time taken for paralysis and death of worms were recorded. Paralysis was said to occur when the worms do not recover in DMSO (0.5% v/v). The worms were declared dead if there was no movement when shaken vigorously in warm water (50°C) associated with change in body color (fading to pale). The results are expressed as mean ± SEM (standard error of mean). Statistical differences were carried out using the analysis of variance and were considered significant when *p* < 0.05 (Table 2).

## 3. Results and Discussion

Table 1 shows the physicochemical properties including calculated molecular weights, percentage yields, melting points, and retardation factor (*R*_*f*_) values and spectral data from ultraviolet−visible and infrared spectroscopy.

Tables 3 and 2 represent results from antimicrobial and anthelminthic screening, respectively.

## 4. Discussion

The antimicrobial activity of these eight synthesised *azo* dyes was determined against seven microorganisms: three Gram-positive bacteria (*Staphylococcus aureus* (ATCC 25923), *Streptococcus pyrogenes* (clinical strain), and *Enterococcus faecalis* (ATCC 29212)), three Gram-negative bacteria (*Salmonella typhi* (clinical strain), *Pseudomonas aeruginosa* (ATCC 27853), *Escherichia coli* (ATCC 251922)), and a fungus (*Candida albicans* (ATCC 10231)) using the high-throughput spot culture growth inhibition assay (HT-SPOTi).

All the eight compounds exhibited antimicrobial activities but to various degree on the six human pathogenic microorganisms. Considering the compound A2 for instance, the chloro group at the para position of the compound A2 was not essential for activity; the substitution of the chlorine group increases lipophilicity, which could have made the molecule get stacked in biological membrane and cause nonbonding interaction with protein groups at the binding site [15]. The esterification of the carboxylic functional in the A1 group to the compound A3 (ester) diminishes activity especially against *Salmonella typhi* and *Enterococcus faecalis*; however, the activity of the ester was greatly improved against *Escherichia coli* by at least eight-fold. The compound A1 had the greatest activity against *Salmonella typhi* and *Streptococcus pyogenes* with an MIC of 62.5 *μ*g/mL followed by good activity against *Staphylococcus aureus*. All the compounds tested showed no activity against *E. faecalis* with the exception of the compound A1, which had little activity with an MIC of 500 *μ*g/mL. The compound A4 (naphthol template maintained with −SO_2_NH_2_: sulphonamide mimic) showed increase in activity against *E. coli* and *Candida albicans* by at least fifteen-fold and onefold, respectively. The presence of the –SO_2_NH_2_ group rather decreased drastically the activity of A4 against the other organisms. Considering the substituents on the compound A1 (carboxylic acid) and A3 (the ester group) (clogP: 5.24 (ChemBioDraw Ultra, Version 14)) are less polar than the carboxylic acid group, which allows it to penetrate lipid membranes. The ester group also makes the molecule behave as a prodrug of which is susceptible to microbial esterases to yield the active carboxylic acid group [16]. Notwithstanding this, the esterases released by the microbes could have degraded the ester hence decreasing the activity, or the ester remains inactive until it is acted upon by the microbial enzymes.

The compound A4 with the sulphonamide group exhibited an activity against *Staphylococcus aureus* with an MIC of 250 *μ*g/mL in the assay. This means that the sulphonamide derivative is more active against Gram-negative *E. coli.* It has been noticed that antimicrobial activities of sulfonamides increases when bulky heterocycles are introduced on their structural templates. For instance, sulfasalazine and sulphamethoxazole are employed in treating various bacterial infections [17]. Overall, the compound A1 was the most active against the organisms, whilst A2 did not show any antimicrobial effect.

With the phenolic *azo* dye derivatives, the compound B4 showed moderate activity against *Salmonella typhi* with an MIC of 125 *μ*g/mL and significant activity against *E. coli* and *Staphylococcus aureus* with MICs of 62.5 *μ*g/mL, respectively. Compounds B1 and B4 showed moderate activity against *Candida albicans* with an MIC of 125 *μ*g/mL. There was drastic reduction in the activity of the compound B3 indicating that the presence of chlorine at the ortho position affects activity. The compound B1 with the carboxylic acid group had reduced the activity throughout against the microorganisms. Esterification of carboxylic acid with the ethyl group diminished the antimicrobial activity completely. Comparing A1 and B1 built on carboxylic acid template, the naphtholic derivative (A1) was more active generally against the organisms compared to its corresponding phenolic derivative.

Overall, B4 showed good antimicrobial activity against most of the organisms with significant MICs. The compound A1 is a broad spectrum; A4 is potent mainly against Gram-negative bacteria with very little activity against Gram-positive bacteria; B4 is also a broad spectrum with little antifungal activity and hence holds potential for future development into novel antimicrobial agents. It can be concluded that the naphtholic derivatives are more potent than the phenolic derivatives in terms of the antimicrobial activity. Nevertheless, none of the synthesised compounds showed good activity against *P. aeruginosa*, which is a well-known notorious Gram-negative organism due to its efflux pumps, resistant genes, and thick biofilm inhibition. *Pseudomonas aeruginosa* is now to be responsible for 65–80% of microbial infections, and hence, more work need to be carried out in structurally modifying this library of compounds to overcome *Pseudomonas*-related diseases [18, 19].

For the anthelminthic activities, it was noticed that the time taken for paralysis and death to occur was increasing as the concentration of the *azo* compounds reduces. It is observed that compound B4 (phenol with the nitro group) had the best activity with low paralysis and death time. It can be observed that the phenolic derivatives of the *azo* compounds had the best activity. It could be said that the naphtholic derivatives have a bulky group as compared to the phenolic derivatives; this bulkiness could have prevented the compounds from reaching their target site, hence, undermining specificity. There is the presence of steric hindrance in the naphtholic derivatives due to the position of the hydroxyl group, which could have affected the binding of the molecule. Therefore, an extra benzene group was not necessary for paralysis of the worms but rather a simple phenolic moiety.

## Figures and Tables

**Table 1:** Synthesised *azo* dye ligands and their physical data.

Compounds	Code	Colour	Molecular formula	Molecular weight (g/moL)	% Yield	Melting point (°C)	*R*_*f*_	UV-vis (nm)	Infrared(cm^−1^)
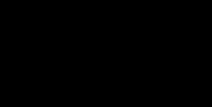	A1	Dark brown	C_17_H_12_N_2_O_3_	292.29	86	240–242	0.84	380	1440 (N=N), 3352 (−OH), 1484 (C=C)
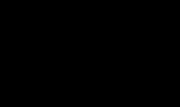	A2	Brick red	C_16_H_11_ClN_2_O	282.72	74	171–173	0.83	250	1451 (N=N), 3300 (−OH), 1573 (C=C)
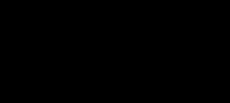	A3	Orange	C_19_H_16_N_2_O_3_	320.34	81	158–160	0.86	360	1418 (N=N), 3612 (−OH), 1574 (C=C)
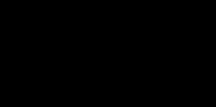	A4	Orange	C_16_H_13_N_3_O_3_S	327.36	88	187–190	0.91	485	1519 (N=N), 3600 (−OH), 1618 (C=C)
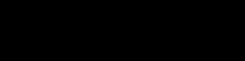	B1	Pale brown	C_13_H_10_N_2_O_3_	242.23	80	240–242	0.74	360	1466 (N=N), 3409 (−OH), 1492 (C=C)
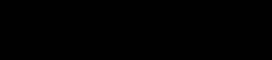	B2	Orange	C_15_H_14_N_2_O_3_	270.28	81	220–221	0.27	360	1418 (N=N), 3612 (−OH), 1574 (C=C)
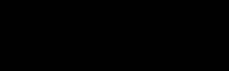	B3	Brick red	C_12_H_8_ClN_3_O_3_	277.66	22	160–162	0.22	250	1451 (N=N), 3600 (−OH), 1573 (C=C)
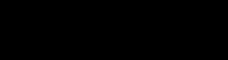	B4	Brick red	C_12_H_9_N_3_O_3_	243.22	79	159–160	0.80	380	1440 (N=N), 3600 (−OH), 1484 (C=C)

**Table 2:** Anthelminthic activity.

Compounds	5 mg/mL		2.5 mg/mL		1.25 mg/mL	
	Paralysis time (min)	Death time (min)	Paralysis time (min)	Death time (min)	Paralysis time (min)	Death time (min)
Negative control (DMSO)	—	—	—	—	—	—
Piperazine citrate	9	10	11	13	24	27
A1	44 ± 2.84	52 ± 2.02	74 ± 1.73	106 ± 2.08	114 ± 2.64	136 ± 1.52
A2	74 ± 0.88	116 ± 0.88	206 ± 3.7	259 ± 1.73	250 ± 1.45	320 ± 2.08
A3	23 ± 2.90	25 ± 2.64	35 ± 3.00	49 ± 2.02	47 ± 1.45	55 ± 0.60
A4	26 ± 0.66	27 ± 0.41	46 ± 1.45	49 ± 1.46	61 ± 0.57	62 ± 2.02
B1	18 ± 0.44	69 ± 2.02	32 ± 1.96	98 ± 2.08	34 ± 0.33	108 ± 0.88
B2	24 ± 0.59	73 ± 1.45	29 ± 1.17	99 ± 1.76	50 ± 2.52	118 ± 2.3
B3	30 ± 0.88	39 ± 1.76	35 ± 0.57	42 ± 2.08	37 ± 0.88	45 ± 2.08
B4	32 ± 0.88	35 ± 0.08	33 ± 0.88	37 ± 1.17	36 ± 0.57	39 ± 0.41

Results are given in mean ± SEM and analyzed by ANOVA ^*∗*^*p* value < 0.05 compared to standard drug.

**Table 3:** Antimicrobial activity of the synthesised *azo* compounds.

Compounds	*Pseudomonas aeruginosa*	*Escherichia coli*	*Salmonella typhi*	*Enterocooccus faecalis*	*Streptococcus pyrogenes*	*Staphylococcus. Aureus*	*Candida albicans*
	Minimum inhibitory concentrations (*μ*g/mL)						
A1	>500	>500	62.5	500	62.5	125	>500
A2	>500	>500	>500	>500	>500	>500	>500
A3	>500	62.5	>500	>500	250	>500	500
A4	>500	31.25	>500	>500	500	250	>500
B1	>500	500	>500	>500	>500	250	125
B2	>500	>500	>500	>500	>500	>500	>500
B3	>500	250	500	>500	250	250	>500
B4	>500	62.5	125	>500	>500	62.5	125
Ciprofloxacin	≤0.5	≤0.5	≤0.5	125	7.8	≤0.5	1
Cefuroxime	>500	250	3.9	500	500	31.25	>500
Amoxicillin	250	>500	125	>500	500	125	15.6
Fluconazole	—	—	—	—	—	—	500

A1–B4 represent the codes assigned to the synthesised compounds as shown in Table 1.

## Data Availability

Data are available from the laboratory of Pharmaceutical Chemistry Department, Faculty of Pharmacy and Pharmaceutical Sciences, KNUST,, Kumasi upon request.
